# E2mC: Improving Emergency Management Service Practice through Social Media and Crowdsourcing Analysis in Near Real Time

**DOI:** 10.3390/s17122766

**Published:** 2017-11-29

**Authors:** Clemens Havas, Bernd Resch, Chiara Francalanci, Barbara Pernici, Gabriele Scalia, Jose Luis Fernandez-Marquez, Tim Van Achte, Gunter Zeug, Maria Rosa (Rosy) Mondardini, Domenico Grandoni, Birgit Kirsch, Milan Kalas, Valerio Lorini, Stefan Rüping

**Affiliations:** 1 Department of Geoinformatics–Z_GIS, University of Salzburg, Schillerstrasse 30, 5020 Salzburg, Austria; bernd.resch@sbg.ac.at; 2 Center for Geographic Analysis, Harvard University, Cambridge, MA 02138, USA; 3 Department of Electronics, Information and Bioengineering, Politecnico di Milano, Piazza Leonardo da Vinci, 32, 20131 Milano, Italy; chiara.francalanci@polimi.it (C.F.); barbara.pernici@polimi.it (B.P.); gabriele.scalia@polimi.it (G.S.); 4 Citizen Cyberlab, Centre Universitaire d’Informatique (CUI), University of Geneva, route de Drize CH-1227 Carouge, Switzerland; joseluis.fernandez@unige.ch (J.L.F.-M.); rosy.mondardini@unige.ch (M.R.R.M.); 5 PM Risk Crisis Change, K. M. Hendrikaplein 57, 9000 Ghent, Belgium; tim@pm.be; 6 Terranea, Bahnhofstr. 120, 82269 Geltendorf, Germany; gunter.zeug@terranea.de; 7 e-GEOS S.p.A, Via Tiburtina 965, 00156, Rome, Italy; domenico.grandoni@e-geos.it; 8 Fraunhofer Institute for Intelligent Analysis and Information Systems IAIS, Schloss Birlinghoven, 53757 Sankt Augustin, Germany; birgit.kirsch@iais.fraunhofer.de (B.K.); stefan.rueping@iais.fraunhofer.de (S.R.); 9 KAJO s. r. o., Sladkovicova 228/8, 01401 Bytca, Slovakia; milan.kalas@kajoservices.com (M.K.); valerio.lorini@kajoservices.com (V.L.)

**Keywords:** social media, crowdsourcing, geospatial analysis, machine learning, image classification, geolocation, 3D reconstruction, architecture, disaster management, near real time

## Abstract

In the first hours of a disaster, up-to-date information about the area of interest is crucial for effective disaster management. However, due to the delay induced by collecting and analysing satellite imagery, disaster management systems like the Copernicus Emergency Management Service (EMS) are currently not able to provide information products until up to 48–72 h after a disaster event has occurred. While satellite imagery is still a valuable source for disaster management, information products can be improved through complementing them with user-generated data like social media posts or crowdsourced data. The advantage of these new kinds of data is that they are continuously produced in a timely fashion because users actively participate throughout an event and share related information. The research project Evolution of Emergency Copernicus services (E2mC) aims to integrate these novel data into a new EMS service component called Witness, which is presented in this paper. Like this, the timeliness and accuracy of geospatial information products provided to civil protection authorities can be improved through leveraging user-generated data. This paper sketches the developed system architecture, describes applicable scenarios and presents several preliminary case studies, providing evidence that the scientific and operational goals have been achieved.

## 1. Introduction

The Copernicus Emergency Management Service (EMS) was established in 2012 to provide information for disaster management in the four phases of the disaster management cycle: mitigation, preparedness, response and recovery. The EMS consists of two components: Mapping and Early Warning [1]. They provide accurate map products based on satellite imagery and monitor and forecast natural disasters like floods or forest fires. However, the experience from recent years of operational service revealed a significant lack regarding timeliness, quality and production capacity [2].

Due to the delay in collecting and analysing satellite imagery, the information requested by EMS users can often be provided until up to 48–72 h after a disaster has occurred. The reasons for this temporal lag are either satellite tasking and orbital constraints, or weather conditions preventing the collection of optical images [3]. Moreover, the crisis maps based on satellite information suffer from quality limitations concerning spatial, spectral and temporal resolution, analysis techniques for damage assessment, usability (e.g., through cloud coverage) and costs for high spatial resolution data [4]. The thematic accuracy of optical satellite-based damage assessment after an earthquake is approximately 65% [5] and that of synthetic aperture radar satellite-based damage assessment after a flood is around 75% [6]. These figures can be significantly lower in urban or vegetated areas. Beyond the quality limitations, satellite tasking reaches its limits when information of a large-scale disaster, covering a large geographic area, is needed, like the earthquake in Nepal in 2015 or the floods in Germany in 2013.

Over the last two years, social media posts have been used in the EMS as an additional layer for disaster maps. Their usage has been steadily increased by manually searching for relevant information in social networks. The use of social media in the EMS workflow was evaluated from April 2015 to January 2017. During this period, the Copernicus Rapid Mapping component was activated 55 times and produced 1200 maps. The evaluation of the Copernicus Rapid Mapping workflow shows that most activations (58%) concerned events with a small magnitude compared to events with a large magnitude (15%). Only in 22% of the activations, a large quantity of social media posts was available, where the number of available social media posts correlates with the severity of the disaster. In 27% of the 55 activations, social media posts were used to complement remote sensing data. As a conclusion, the majority of the available social media posts have not been considered in the EMS workflow, although they include valuable information for disaster management and are continuously increasing.

Other disaster management activities than EMS complemented existing systems and procedures throughout the last years with crowdsourcing initiatives (see Section 2.1). Like this, various types of crowdsourcing activities have demonstrated that the availability of internet technologies and online communities are of great value to disaster management. Mostly, user-generated data can fill information gaps by combining human intelligence together with artificial intelligence, improving disaster management capabilities for a variety of labour-intensive manual tasks through employing digital volunteers [7].

The outlined shortcomings of the current EMS workflow and the potential of social media led to the design of the research project “E2mC-Evolution of Emergency Copernicus Services,” funded by the European Commission through its Horizon 2020 programme [8]. The project’s goal is to demonstrate the technical and operational feasibility of integrating social media and crowdsourced data into the EMS Mapping and Early Warning components. Therefore, a new EMS service component (Copernicus Witness) designed to exploit social media analysis and crowdsourcing capabilities to generate a new information product, ensuring the availability of relevant information in near real time. More concretely, the current remote sensing data based system is extended by semantic, temporal, spatial, image and video analysis algorithms for social media posts and crowdsourced data, which are collected by remote digital volunteers and local eyewitness reporters. The motivation of volunteers to participate in our crowdsourcing activities and to share information about a present event is a key element in this project and underpinned by recent publications [9,10,11]. The social media and crowdsourcing analysis results are then combined with satellite data based map products to enhance information quality and reduce the time for producing reliable information.

The objective of this publication is an in-depth discussion about an architecture with its components that combines geospatial, temporal, semantic, image and video analysis methods applied on social media and crowdsourced data with the focus on natural disasters. To the best of our knowledge a comparable architecture has never been published. The presented system is not restricted to natural disasters but can also be applied on other large-scale events like man-made emergency situations and humanitarian crises. Furthermore, the discussion of the single components raises several research questions that will be tackled in the scope of this research project. Additionally, an advantage of our approach in comparison to other is the combination of various data sources that lead to an improvement of data quantity (using various social media sources) and quality and consequently improved analysis results.

## 2. Related Work

Social media data has been used by research groups and companies to provide additional functionalities to authorities, crowds, etc. during a disaster event [2,3,4,5]. A small selection of social media platforms is compared with the proposed component in this section. Furthermore, current research efforts in analysing text, image and video are described in a geospatial context.

### 2.1. Social Media and Crowdsourcing Platforms in Disaster Management

The platform “Tweak the Tweet” [12] enables people to communicate with authorities in a two-way communication channel over Twitter during emergencies, crisis and disasters. The communication follows a defined syntax and descriptive hashtags formalise the communication. Due to the formalised messages, the tweets of the crowd are collected with minor effort by filtering. The next steps involve extracting geolocation information, creating incident reports as well as sorting them into five categories. Finally, the processed tweets are displayed in various forms in a web-application.

The open-source platform “Ushahidi” [13] was developed to collect and visualise crowdsourced reports of violence to raise awareness of predators. People can contribute via SMS, mobile apps, Twitter, email and other channels. The received reports can then be visualised on charts, graphs and maps. The Platform is steadily expanding and is an excellent example of successful crowdsourcing and its possibilities.

“Pybossa” [14] is an open-source platform for crowdsourcing projects which requires human intelligence (e.g., image classification, describe the content of a video). The project creator determines the tasks with different customisation possibilities like demanding that a task has to be completed by more than one person. This platform was used in the context of the typhoon Pablo which hit the Philippines in 2012.

Lastly, the open source platform “MicroMappers” [15] enables people to contribute voluntarily during disasters by classifying images, videos and aerial photographs via tweets or SMS. The information is consecutively analysed and geotagged. This platform was actively used when Hurricane Matthew hit areas across the Western Atlantic in 2016, collecting over 250,000 images and six million text messages.

The proposed architecture in this paper outlines how many of the techniques and methods used by the described platforms in this section, can be integrated into one service. Additionally, most of the described platforms only provide a single functionality and consider data from one or two social media networks, whereas the proposed component exploits various data sources and functionalities.

### 2.2. Topic Modelling of Short Texts

Algorithms that extract information from a text can be divided into clustering and classifying algorithms, which are referred to as supervised and unsupervised in machine learning. Classifying algorithms are trained on a set of training records that are labelled according to a finite number of classes [16]. Multiple supervised text classification algorithms are described in the literature [17,18,19,20,21,22]. The labelling task of the data can cause a tremendous effort as a significant amount of data is needed and can be an impossible task due to the sheer amount of the data. Another possibility would be a semi-supervised algorithm that uses labelled and unlabelled data. Such an algorithm can achieve a higher classification accuracy in various applications but it can also decrease the performance [23].

In contrast to supervised algorithms, unsupervised algorithms rely solely on the given data. Traditional clustering algorithms include hierarchical clustering algorithms [24] and partitional clustering (i.e., k-means) [25]. Another way of clustering is using probabilistic topic models that cluster words and documents simultaneously [16]. Well known topic models are Latent Dirichlet Allocation (LDA) [26] and Probabilistic Latent Semantic Indexing (PLSI) [27]. The compelling topic model LDA produces a mixture of topics for every document with probabilities providing information not only on the document level but also on the topic level.

Due to the restricted content length in social media posts like tweets, where a post cannot exceed a 140-character limit, standard text mining approaches cannot be applied to their full potential. An approach by [28] is to aggregate all posts of a Twitter user to ascertain specific topics that users share information about. The topics are then compared with topics of their followers to determine if users follow others because they publish tweets about similar topics. A similar approach is carried out by [29], where two kinds of topic models are compared: the standard LDA model and its extension, the Author-Topic model [30], where the tweets are aggregated for the analysis. The results showed that the Author-Topic model does not yield a better performance than the standard LDA. These approaches conduct experiments with conventional topic models and show a limitation in analysing short posts. Instead of analysing every tweet, they aggregate them depending on the publisher.

A topic model for short posts was introduced by [31], where the model is trained by identifying word co-occurrences in posts (i.e., biterms) and uses the aggregation patterns on the whole corpus to overcome the sparsity problem on the document-level. Another approach [32] incorporated meta information of Twitter in their topic model. As a result, the topics do not only consist of words but also of hashtags, which can be used to label the topics. However, both of them have hardly been tested in real-world-scenarios.

### 2.3. Geospatial Analysis of Social Media Data for Disaster Management

Spatial autocorrelation can be applied on various spatial data sets (e.g., georeferenced social media data) to identify spatial dependencies concerning social-economic phenomena. Statistical tests assess if near-by data is similarly classified and show associated topic indicators [33]. The most frequently applied statistical tests are Moran’s I [34,35], Geary’s C [36] and Getis-Ord G [37] that quantify Tobler’s first law of Geography: ”Everything is related to everything else but near things are more related than distant things” [38]. Global autocorrelation statistics (e.g., global Moran’s I statistics) measure the overall dependency of the complete data set, in contrast to local spatial autocorrelation (e.g., local Getis-Ord G_i_* statistics) which can be used to identify clusters within the data set. For a hot spot analysis, local measures are required to determine positive (hot spot) and negative (cold spot) *z*-scores and small *p*-values in a data set [39].

Local spatial autocorrelation has been used in various real-life applications. It has been used to detect patterns of traffic collisions [40,41]. In another publication, the spatial patterns of acts of terrorism were assessed at country level [42]. Hot spot analysis were applied in numerous other applications [43,44,45]. A hot spot analysis based on social media data is being performed by [46,47,48].

In recent publications, new data sources like social networks were added to disaster management workflows providing additional information for emergency managers [49,50,51,52,53,54,55,56]. A comprehensive review about social media communication in disasters was published with a framework demonstrating various entities that utilise and produce disaster social media content [49]. Real-time crisis maps were provided based on social media data [50]. The social media data were geocoded with locations from gazetteer, street map and user-generated data and reached remarkable results in comparison to post-event impact assessments from national civil protection authorities. Statistical analysis methods were applied to identify flood-related tweets in the context of disaster management [51]. Their results show that georeferenced tweets in proximity to the occurred flood have a higher probability to be flood-related than other tweets. In another publication, preliminary results were shown in classifying social media data into stages of disaster management (mitigation, preparedness, emergency response and recovery) by manually annotating tweets and training a logistic regression classifier [52]. These results are helpful for emergency managers to follow the transition of the disaster in its four phases. Multiple non-authoritative data sources were used to improve flood assessment [54] and the Twitter stream was monitored to detect earthquakes [53]. Geospatial systems have also been used to improve the situational awareness during a disaster by analysing the retrieved social media data [55,56].

The aforementioned publications demonstrate preliminary results concerning the geospatial analysis of social media data in disaster management workflows and show that there is a need to conceptualise the various methods in a single component.

### 2.4. Geolocating and Classifying Images

Computer vision algorithms can calculate image features for comparison but are demanding in computational power. Available algorithms can determine colour, shape and image texture [57]. However, the challenge is to model high-level image semantics like feature descriptions. The research areas machine learning and artificial intelligence provide new ways to solve complex problems like these [58].

Besides the image and video content, the spatial location of media is a fundamental component for mapping the results. The IPTC Photo Metadata Working Group tested in 2013 and 2016 how social media sites manage metadata embedded in images that are uploaded to the sites [59]. The results show that widely used portals like Facebook and Instagram are stripping off all metadata. There exist also dedicated photo and video sharing websites like Flickr or YouTube that allow the geotagging of photos and videos.

The automatic estimation of the location of images is computationally challenging. Some authors use textual features like tags and image title as well as the description or comments posted by other users to estimate the location of images [60]. For example, a data-driven scene-matching approach was applied comparing single images including their colour and edge histograms and geometric context with an evaluation data set of more than six million Flickr photos [61]. In another project, user related traces on Twitter were integrated to estimate the location of photos shared by this user [62]. Other studies investigate the matching of candidate photos with known landmarks retrieval focus by exploiting location-aware visual features [63].

Besides the attached image, the describing text can also be analysed to disambiguate the location of the social media post. The feasibility of geolocating images shared on Twitter posts was shown by extracting implicit geographical references from the text of the tweet and highlight a correlation existing between text features and image features [64]. Besides the challenges related to the usefulness of the extracted locations, like their precision, accuracy and credibility, the extraction phase introduces errors (false positives and false negatives), due to ambiguities which exist between location names and other proper or common names [65]. To overcome these problems, the social network of a post is taken into account to obtain an additional context potentially useful in overcoming ambiguities, as pointed out in [66]. This privileges as well the extraction of locations related to the target event [67].

### 2.5. 3D Reconstruction from Videos

Structure-from-Motion (SfM) allows high-resolution modelling of dense point clouds and reconstruction of 3D surfaces [68]. Similar to stereoscopic photogrammetry, the technique uses a series of overlapping images to reconstruct the 3D surface. Matching features from a set of multiple images are automatically extracted leading to the triangulation of camera position and relative object coordinates. Through an iterative process, multiple point meshes are refined to a final 3D point cloud that can be overlaid with texture from the original images. The relative coordinates have to be transformed into an absolute coordinate system through ground control points [68].

3D-reconstruction is for example used to build very high-resolution digital elevation models from unmanned aerial vehicle (UAV) imagery or for the inspection and visualisation of buildings and infrastructure as 3D models allowing their viewing and inspection from all sides. Successful approaches were also made to create 3D image scenes using unstructured images from the Internet [69,70].

In the disaster context, 3D reconstruction allows assessing disaster damages from ground-based photos or UAV imagery. A before-after comparison of damaged buildings in the aftermath of a disaster event allows better estimation of the severity of damages and estimations of the cost of reconstruction.

## 3. Scenarios

The following scenarios illustrate the new functionalities added to the EMS system by developing the new Witness component. In these scenarios, the EMS operator can evaluate the current stage of an event with statistical tools by filtering the data with various configuration parameters. Furthermore, the data themselves are ranked according to their reliability by an automated algorithm and as well as manually by the crowd. The operator can interact with the crowd by creating hashtags and tasks.

### 3.1. Early Activation

As discussed in previous sections, a severe shortcoming of the current EMS system is the time delay of the first produced maps of the area of interest (AOI), which shall be reduced from 48–72 h to approximately 24 h through the Witness component.

First, abnormal activity in social networks is detected automatically by analysing social media posts on the basis of pre-defined criteria (e.g., keywords, clustering, ranking). A continuously running crawler collects new social media posts, which are analysed in pre-defined intervals. This monitoring process requires action of the EMS operator or the occurrence of a current disaster.

Furthermore, E2mC will provide the possibility for EMS operators to filter streams for disaster-related data in order to visualise relevant statistical information (e.g., temporal peaks, geospatial heat maps, different graphs) and to identify representative keywords and hashtags of a disaster. Like this, the EMS operator will be able to identify the AOI with a high certainty and can trigger the satellite tasking for the identified area.

### 3.2. Improvement of Social Media Data Usage

An objective of the Witness component is to improve the quality of social media analysis results. This has an impact in the different phases of the EMS procedure: in the early mapping phase before the arrival of Earth Observation (EO) data arrival and in combining social media posts and EO data for accurate mapping. Here, the main challenge is to filter the data by available metadata (e.g., geolocation, user, language) in order to reduce the false positive selection of tweets (e.g., users who are not impacted by the disaster). This can be accomplished only if the information derived from social networks is instantly processed and its reliability is confirmed.

**Figure 1:**
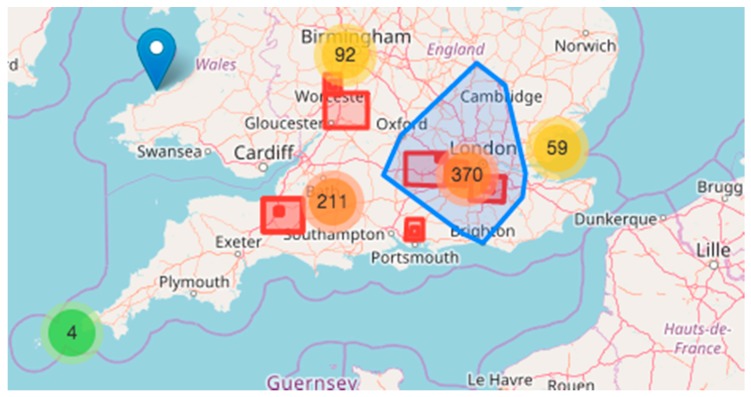
Social crisis map for 2014 Southern England floods–the areas in red refer to maps produced in the EMSR069 Copernicus rapid mapping activation; tweets are clustered based on their geographical location which is visualised as the absolute number of tweets containing an image. The clusters, represented as orange/yellow/green circles point out the size of the cluster from largest to smallest; by clicking on a number, all underlying tweets are displayed together with an outline of the area in blue.

Thus, the incoming data are automatically ranked according to their relevance. An example for high-ranked information would be a tweet referencing an image with geolocation information in the determined area of the disaster, from a known source (e.g., official Civil Protection of the country). The geospatial information can be used by a geographic information system (GIS) to extract relevant information, e.g., add the georeferenced or geolocated (see Section 4.6) images to a GIS layer to visualise information about damaged buildings or flood marks. Figure 1 shows the distribution of geolocated Tweets containing images for the Southern England flood in 2014. The rectangles show the areas for which rapid maps were produced, while the polygon shows the area referring to number 370 in the map. Another example for high-ranked data are georeferenced videos from known sources that can be visually analysed.

Furthermore, a warning map is created based on the data filtered according to pre-defined criteria such as the exclusive use of trusted sources. Using a visual interface, the EMS operator has the possibility to filter the results (e.g., by date, geolocation, full-text, tags, ranking, presence of image/video, various categories and in general all those applied by automatic tools), to visually evaluate the results (view images, interpret social media posts, etc.) and to adapt the warning map accordingly. Moreover, the analysis results can be provided via the web to authorised users (e.g., civil protection agencies).

### 3.3. Leveraging the Crowd for Social Media Filtering

Crowdsourcing will leverage the quality of social media data through human intelligence. The crowdsourcing component can be triggered by an automated procedure that measures the accuracy of the data classification. Crowd tasks can then be created and shared to validate the results. Furthermore, tasks can also be created in the preparedness phase to increase the number of labelled social media posts for improving the accuracy assessment. The major goals of this crowd tasking approach are to clarify if the content of the social media data can be geolocated and if the social media data include information to delineate the AOI or damages (e.g., flood marker, damage marker).

In the case of a disaster triggering enormous response on social media channels, the number of relevant social media posts is too extensive to be handled by disaster managers. In this case, the operator can create crowd tasks to filter the contents. The results of the task performed by the crowd (e.g., filtered links) are stored on the Witness component and are shown in the GIS environment (see Section 3.2).

### 3.4. Leveraging the Crowd for Micro-Mapping

During severe disaster events like the earthquake in Nepal 2015 or hurricane Matthew in 2016, when large geographic areas are impacted, it is difficult for the EMS service to provide all required maps in a short time. In this case, the EMS operator can activate and coordinate a specific crowd of volunteers, selecting from a list of available mapping crowds. The EMS operator can control the task assignment as all the information of the crowd and the EMS is accessible to them. Additionally, the EMS Witness component aims to leverage the results of other available volunteered mapping efforts like the Humanitarian OpenStreetMap Team (HOT) and TomNod that commonly auto-activate themselves. For this purpose, the EMS operator maintains information (contacts, website) of existing crowdmapping initiatives.

Ideally, the EMS Witness component does not provide a separate, alternative platform for crowdmapping but it relies on existing operational platforms. Moreover, it is important for EMS to spread the use of crowdmapping to cover small-scale events as well.

### 3.5. Crowd Engagement

Highly valuable information can be retrieved from crowdsourcing efforts of people who are directly affected by a natural disaster. These people can provide valuable in-situ information about the impact of an event and contribute to a faster creation of disaster maps. In this case, the EMS can use social media as a channel for collecting information. To foster focused information generation, the EMS operator can define a disaster-specific hashtag, let the crowd share information through this hashtag and push the generated content to the local citizens. This can happen only if authorized by the civil protection as it is the official channel for the communication with the citizens. The hashtag is shared via official channels (e.g., civil protection) with the local crowd.

The EMS will provide a responsive web app for uploading crowdsourced information, but the relevant type of information depends on the kind of disaster. For instance, in the case of both earthquakes and floods, georeferenced images of damaged buildings are required, whereas in the case of floods georeferenced text, describing where a flood happens, may also be enough.

## 4. Architecture for Leveraging User-Generated Data in Event Detection and Disaster Management

This paper proposes an infrastructure to exploit social media analysis and crowdsourcing capabilities to generate information layers that are relevant to disaster management in near real time. More concretely, current remote sensing data based approaches are combined with semantic, spatial, temporal, image and video analysis of social media and crowdsourced data. The long-term goal is to integrate the developed Witness component into the Mapping and Early Warning systems of the EMS.

### 4.1. Overall Architecture

**Figure 2:**
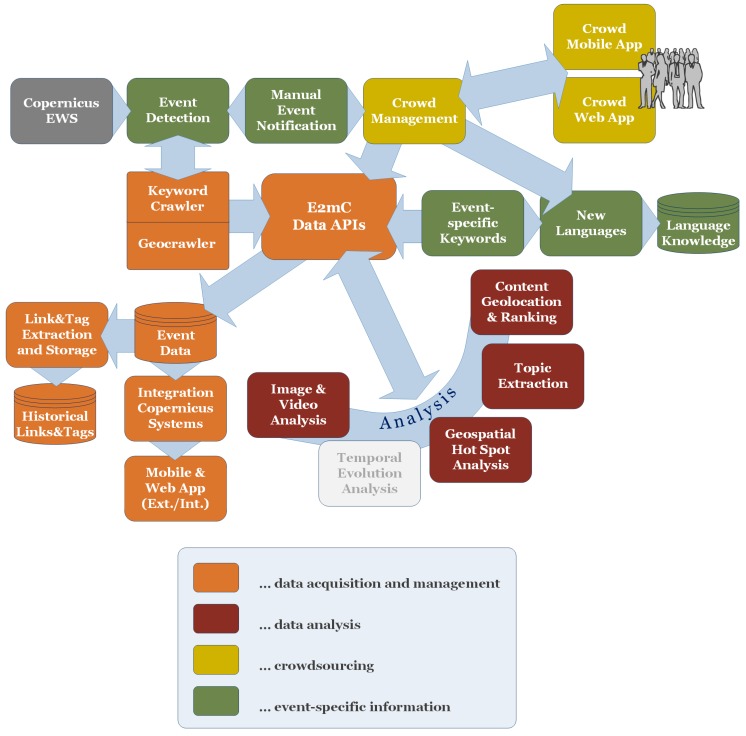
Functional Architecture of the Witness Component.

Figure 2 illustrates the general architecture of the EMS Witness system. It comprises components for data acquisition, storage, management and analysis and graphical user interfaces for the EMS operators and the crowd. Understanding the interaction between the different components and their order in the workflow is crucial to grasping the selection of the components.

The workflow is triggered either by the EWS component in case of a flood or manually by an EMS operator. Various parameters have to be selected for the different analysis components and the crawlers before start. In case of a flood the EWS component provides information including probability, timing, location and magnitude of an event. The EMS operator chooses the parameters for Witness workflow and can use the parameters obtained by EWS. When the EMS operator starts the Witness workflow, crawlers begin to collect social media posts. The collected data are stored via a centric RESTful API with six resources e.g., user, event, keyword, post, image and tag in a relational database model, which is not discussed in this paper due to it simple nature according to the state of the art. The API is the access point for the components to request data and store results. The components analyse the geospatial, temporal, semantic, image and video content. Furthermore, a campaign is started to enable micro-tasking for the disaster event. The georeferenced images and a disaster map, based on georeferenced text, are visualised in a web app where the operator can query valuable information for disaster management. In the next sections the main components of the architecture are described to give an insight of the methods integrated into the components.

### 4.2. Early Warning: Alert of an Event

The EWS component is based on two early warning systems: European Flood Awareness System (EFAS) and European Forest Fire Information System (EFFIS). The focus in E2mC is on the EFAS that provides operational flood predictions in the main European rivers up to 10 days in advance including information on event probability, timing, location and magnitude. The service has been fully operational since 2012 and is available to hydro-meteorological services with responsibility for flood warning, EU civil protection and their networks. EFAS forecasting products are available twice daily. In addition to routine flood hazard forecast, EFAS will also provide rapid assessment of the associated risk.

The AOI are initially defined focusing on potentially flooded areas identified by the EFAS system. Even though the prediction skills of EFAS are constantly improving and provide already a good indication about the location and timing of the flood, EFAS information is still highly uncertain in case of extreme events. Once a critical threshold either on flood hazard return level or risk indicators is exceeded, the system will automatically generate initial set of AOI, event-specific metadata and make them available to other components. The following social media analysis components try to confirm and validate EFAS’ result to enable the EMS operator to make well-informed decisions on satellite tasking.

### 4.3. Text-Based and Geospatial Crawling of Social Media Posts

Social networks like Twitter, Flickr, Foursquare, YouTube, Facebook and Instagram provide collected data, i.e., social media posts, via Application Programming Interfaces (API). These APIs are used to harvest a variety of attributes, which are sometimes specific to a social network and partly generic, including post’s ID, text, geolocation, date or a username. These attributes have proven valuable for disaster management.

The **Geocrawler** component requests data for a predefined AOI for all of the mentioned social media networks. The area is defined either through an automated process (see Section 4.2) or by the EMS operator. Due to the restrictions by the social network APIs with respect to the allowed number of requests per time unit and the maximum number of data sets returned per request, the Geocrawler follows an intelligent algorithm to minimise the number of requests: The AOI is dynamically divided into smaller polygons (rectangles, hexagons) depending on the number of returned data sets, i.e., the target region is split into smaller sub-regions if the number of returned data sets exceeds the limit of the particular API.

The **keyword-based crawler** requests social media data according to expressions that are defined by the EMS operator, stored in the multi-lingual “language knowledge database” (see Section 4.5), or defined by the EWS component. The crawler then filters social media data by comparing the keywords with the textual content of the posts. Through keyword-based filtering, this crawler can be used to continuously monitor the activity in social media networks concerning natural disasters. The frequency of particular words associated to disasters can be temporally analysed to detect a new or upcoming disaster.

### 4.4. Semi-Supervised Topic Modelling

The language used in social media networks changes rapidly as new hashtags are continuously defined, or new abbreviations are introduced. Therefore, an algorithm analysing this kind of unstructured and unedited text in social media needs to be capable of dealing with these irregularities. Thus, E2mC includes the development of a semi-supervised machine learning algorithm to identify relevant words and topics in social media. The results are evaluated by comparing them with pre-labelled data.

Before analysing social media posts, a number of pre-processing steps has to be applied in order to filter statistical noise, as demonstrated in [71]. The following pre-processing steps enhance analysis performance and are shortly summarised herein. The order of the pre-processing steps is essential to achieve the best possible results. This particular order of pre-processing steps and methods is chosen to achieve the best results for English language and may have to be adapted for other languages.

In the first step, tokenisation is performed where the text is split at every blank character to create a list of single tokens of the character stream. Characters without semantic meaning are removed, including commas or semicolons. However, some punctuation marks may carry relevant content, e.g., exclamation marks underpin the importance of a statement [72]. Tokens are then converted to lowercase to decrease the influence of typos. Yet, it is also plausible to incorporate the special meaning of words that consist of capital letters only, denoting the particular importance of an expression [73]. Furthermore, URLs, numbers, special characters are removed which are considered noise in the text for the particular setting of our research. Then, synonyms are compressed into single words (e.g., “quake,” “eq” or “shake” are integrated with the word “earthquake”), which is an important step to increase the significance of the outputs. Currently, this step still requires a manual list of synonyms. Automating the process of creating this list through a machine learning algorithm is subject to future work.

In the following steps short words (less than three characters), stop words (e.g., “the,” “do”) and unique words (only appearing once in the text corpus) are removed as they do not add valuable semantic information for this task, according to [74]. Then, stemming is applied to reduce the words to their root [75], leading to better results for the applied bag of words approach as described in the following paragraph. The stemmer works for various languages (English, Russian, Italian, German and others) [76].

**Figure 3:**
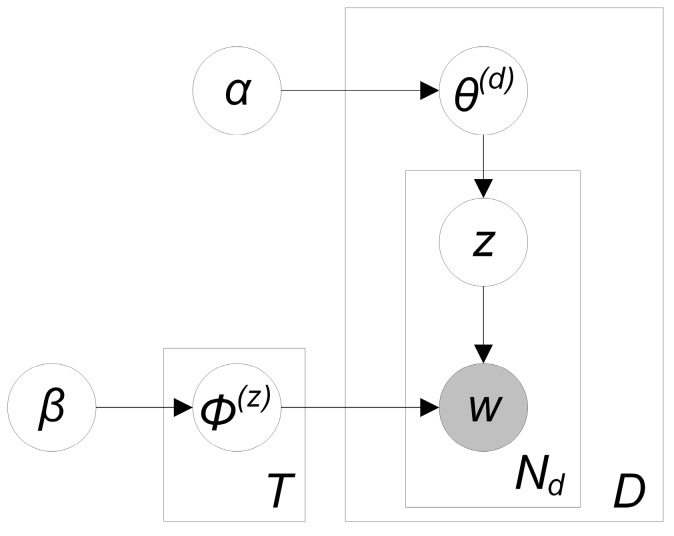
LDA plate notation [74].

In the next step, the topic modelling algorithm LDA [26] is applied to extract semantic topics from the pre-processed social media data. LDA requires a number of input parameters (see Figure 3). First, *T* represents the number of topics to be generated from the documents D (i.e., social media posts). Second, *α* influences the document-topic distribution. In fact, *α* increases the sparsity of the topic distribution when it is close to zero, whereas it smooths this distribution when assigned a larger value [77]. The parameter *β* represents the distribution over words in a topic, meaning that a topic contains more or fewer words depending on the value of *β*. Like *α*, *β* also smooths the distribution when assigned a higher value and the distribution becomes sparser when assigned a lower value. *φ, θ* and *z* are latent variables that are inferred during the process and *w* represents the observed words of the social media posts.

The processed social media posts originate from a number that differ in text length. For instance, a Facebook post can reach several thousands of characters whereas Twitter posts are limited to 140 characters. To account for this matter, it is recommended to adapt the prior variables for every social media source. Two specific LDA variations that tackle the problem of short documents are shortly explained in Section 2.2. More information about LDA and its parameters can be found in [26]. In the Section 4.10, the accuracy assessment of the topic model is explained.

### 4.5. Language-Independent and Language-Specific Topic Modelling and Information Extraction

The goal of the multi-language management component is to provide the capability to quickly set up a new component for topic extraction and identification of relevant social media posts. It aims at enabling users without knowledge in social media analysis to extract relevant content from emergency-related post, written in an untested language. The component therefore implements a partly language-specific and partly language-independent workflow consisting of the following steps: translation of keywords, data processing, model training and parameter tuning, identification of relevant topics, evaluation, model selection and model application.

During the translation process, relevant keywords are translated into the specific language. Synonyms, language- and scenario-specific words, such as location names or hashtags can be provided through crowd activation. Additionally, an automatic translation is performed by requesting the Microsoft Translator API. The identified keywords are stored in a language knowledge database and will later be integrated in the preprocessing and model training phase. Apart from that, a language-specific keyword list is provided to the crawling component in order to extract scenario-related posts.

Following the translation, the social media data provided by the crawlers is currently transformed into a bag of words matrix in the text processing phase. As described in Section 4.4, the training of topic models includes a variety of pre-processing steps such as the tokenisation of words, stemming and the removal of stop words. Most of these steps need to take into account the characteristics of the written language to reduce the noise by recognising stop words, synonyms, abbreviations and word boundaries. Thus, it is usually not possible to use the same pre-processing steps and the same order to process texts in previously untested languages [78]. For each language, different rules have to be applied to detect tokens with respect to language-specific word boundaries. A common approach to remove stop words is to provide a stop word list for each language. A more sophisticated approach to treat stop words in topic models is presented in [79] by setting specific hyperparameters.

We combine the two approaches and provide a stop word list for the most common languages. In case of an uncovered language, words are filtered by their frequency. Additionally, language-specific tokenizers will be applied and synonyms for emergency-related words, such as “flooding” and “inundation,” provided by the crowd, are replaced by the basic word, in this case “flood”. Creating a stop word list based on machine learning refined with integration of the crowd will be part of the future work.

Language-dependent processing is not only necessary to transform and prepare the content but also during the model generation. The hyperparameters used in the training process can have a high influence on model performance [79]. Thus, in every upcoming event it is necessary to adjust the pre-processing and training workflow with respect to the given language and scenario. To select and realize the most efficient workflow, several topic models are trained by orchestrating pre-processing steps and applying different combinations of hyperparameters. In order to reduce the hyperparameter search space and with that the number of trained topic models, we try to identify regions in the search space in advance with the most promising results based on experiments on test datasets.

Following the preprocessing and hyperparameter tuning, an additional challenge constitutes the identification of topics extracted in the training process in a new language. In order to identify topics of interest, such as a topic referring to damage on infrastructure, language- and domain-specific knowledge must be provided. One method is the manual analysis of the words appearing with high probability in the topic-word distribution, another automatic approach is based on matching keywords. For example, crisis-related social media posts are identified and classified in 23 information-specific categories providing a keyword dictionary which could be also used for topic identification [80]. In this scenario, the keyword-based approach faces difficulties, since it requires the translation of the keyword list for every new language and scenario.

Another approach to facilitate the topic identification without the need for translation is the integration of domain- and language-specific knowledge during the model training. An interactive topic modelling method is proposed in [81] that provides the possibility to define constraints in form of word pairs that have to appear together in one topic. In another publication domain knowledge is integrated by defining a target keyword and present an extended LDA algorithm that allows to model topics around this target aspect [82]. This method is named Targeted Topic Modelling and it forms the basis for the approach implemented in this component to extract content. According to the Targeted Topic Modelling approach, the focus is set to an aspect the user is interested in. A target is represented by keyword list which is the basis for the topic model generation. The extracted topics are represented by a major word distribution for the irrelevant topic and multiple word distributions for the relevant topics. To assign relevance to documents during the assumed generation process, a relevance status is sampled for each document. This relevance status depends on whether the document contains relevant targeted words or not. When the document is assigned the relevance score 0, the words are sampled from the major irrelevant word distribution. When the document is assigned the relevance score 1, the document generation is similar to the basic LDA Assigning a relevance score to each social media post enables to identify scenario-related social media posts, while the assigned topic distribution allows to further categorize the emergency-related content. In order to cover a rich spectrum of relevant information and to include more information relevant for the identification of topics, we extend the Targeted Topic Model and enrich the input data with additional keywords such as locations, hashtags, handles and links.

After several Targeted Topic Models are trained with different hyperparameters, in the evaluation phase the model performance is evaluated and the model providing the best overall performance will be applied further. This evaluation of topic models is an often-discussed challenge that in most cases includes a manual analysis of the generated topic word distributions.

To automatically evaluate a trained model, [83] estimate the probability for held out and previously unseen datasets. If with respect to the extracted topic word distribution the probability for the unseen document is high, the model will to generalise on unseen data. Although this is a language-independent approach, several analyses in this field show that the results do not always agree with what humans identify as coherent during a manual analysis. Another publication proposes to assess the topic coherence to humans by comparison with other information sources such as Wikipedia [84]. Since this approach is more challenging when dealing with data in different languages, we focus on evaluation metrics allowing to integrate knowledge provided by a local crowd. In [85] this is achieved by two different tasks presented to a crowd, the word intrusion and the topic intrusion task. Word intrusion means extracting the top-n words from a topic and switching one word with a word from another topic. If this outlier can be easily detected by a human, the topic is evaluated as more coherent. This evaluation process can also be executed automatically to reduce the crowd effort [86].

In order to select the model with the highest performance, we combine the above-mentioned approaches. To initially reduce the number of eligible models, the first validation will be performed automatically based on available structured data, such as the location names from the region of interest or the information contained in attached images. Depending on the occurrence of this information in the documents, coherence and usefulness of the assigned topics can be assessed. In a second step, this reduced set of models is evaluated by presenting the model results to the crowd, letting them label relevant groups of social media posts, and through the above explained word intrusion and topic intrusion tasks.

Following the selection of the model with the highest performance, in the model application phase the model is applied to extract relevant content. The assigned relevance status per posts is used to identify emergency-related posts and after that they will be categorized based on the assigned topics.

### 4.6. Content Geolocation and Ranking

The locations mentioned in social media posts can be at different levels of granularity (e.g., city or region) and quantity (i.e., once or multiple times mentioned). For supporting the rapid mapping activity, the goal is to identify images related to precise locations (streets or points of interest) in the AOI. However, experts would also benefit from imprecise locations, as they could locate an image even with an imprecise location in an AOI.

The network-based geolocating algorithm proposed in [67] selects a set of candidate locations for each social media post, identified as the n-grams which potentially refer to a location. In the current implementation, they are obtained using high-recall Named-Entity Recognition (NER) [87] with multi-language capabilities [88,89] but there are alternatives as well, such as n-grams matching predefined patterns. Then, the algorithm tries to disambiguate candidate locations by building a local context, i.e., searching for geographical correlations among candidate locations. External geographical information is needed for this purpose and in the first experiments OpenStreetMap (OSM) [90] as well as GeoNames [91] were used. While GeoNames allows locating localities and points of interest (POI), OSM has the advantage of introducing more fine-grained information, with precise locations for a large number POIs and at street level. On the other hand, the results may be noisier using OSM as names of locations may appear also in names of, e.g., restaurants or streets, so further disambiguation is needed. Therefore, only the locations for which it is possible to find a correlation are disambiguated, while the others are considered ambiguous. This technique is traditionally used for longer and context-rich documents, like web pages but the short and decontextualised nature of social media posts tends to reduce its effectiveness. In the productive system, information from OSM and GeoNames is downloaded and maintained before and after an event in order to provide up-to-date information in case of an emergency.

In a first case study (Central Italy Earthquake in August 2016) less than 4% of tweets had locations that can be disambiguated by building a local context. Therefore, the algorithm was extended by connecting social media posts in a behavioural, social network, i.e., a social network based on implicit interactions among posts rather than specific relationships among users [92]. In particular, social media posts are connected if they share similar content or belong to the same conversation (e.g., following reply or retweet links or using the same hashtags). In this way, a global context for a social media post is built from the social network. The global contexts overcome the limits of the local contexts, allowing the disambiguation of their location or, alternatively, the inference of a related location if no locations are explicitly mentioned in the text of the social media post. Using the global context, the algorithm is able to disambiguate locations in more than 20% of tweets with a precision >90% in the Italian earthquake case study (using GeoNames as a gazetteer and locating tweets at the level of locations or POI). The algorithm is currently under development to further improve performance. Even if improving recognition, disambiguation and inference reduce geolocation errors, false positives/negatives cannot be totally avoided and represent an issue that can be successfully tackled with crowdsourcing, i.e., letting the crowd manually validate the proposed geolocation of social media posts.

As the number of social media posts and images to be examined for mapping purposes is too comprehensive (see Figure 1), there is a need for ranking the social media posts containing images, in order to present to the mapping operators the social media posts with higher relevance first. Geolocated content is therefore ranked based on the following factors:

**Location precision**: the location can be obtained from georeferencing or geolocating images on the basis of textual information, at different levels of granularity, as discussed above.

**Timeliness**: images may lose relevance over time and images preceding the event being considered are not carrying new information.

**Trust of source**: the source of information has an importance in the ranking, i.e., the more trusted the source is, the more complete and reliable the provided information will likely be.

**Usefulness**: the usefulness of images for mapping tasks, i.e., if they provide additional information.

The given criteria are combined in a formula with a numerical indicator to evaluate the rank of the geospatial information. The indicator can be a basis for ranking images in visualisation tools, to be able to show and give priority to images that are likely to provide precise and useful information. The above metrics should be used in combination. The ranking formula is defined as follows:

rank (img,loc)=best (location_precision)×trust_of_source×usefulness

Each image is associated with a rank for a given location extracted from a social media post. If several locations are associated with possible locations for the same image, each pair <img, location> is associated a different rank. All metrics are defined in the [0, 1] range.

When considering location precision, precedence should be assigned to geotagged images. However, since their number is likely to be a small percentage if extracted from social media posts, a metric for location precision for geolocated images is needed. Assuming that the geographical hierarchy of locations such as in GeoNames [91] is adopted, the precision can be based on a parameter combining the obtained level in the hierarchy and the confidence in the result. For locations based on OSM, we distinguish between locations at locality level and POI or street level. In case several hypotheses can be made (e.g., when the same image is found in different social media posts), confidence can be expressed by evaluating the percentage of compatible locations found for the same location.

Timeliness depends on the time at which the information is ranked, the starting time of the event and the time associated with the image, if available (metadata for images in tweets are not available, so the only source of information is the date and time of the tweet). If time is not available, we associate to each image the earliest time in which the image is found in a tweet in the available set of tweets. If the time associated with the image is before the event of interest, timeliness is null. Otherwise, we assume to select only images for the interval of interest for the mapping activity and that all images are ranked the same way if related to that interval.

Trust of the source is introduced in the ranking to differentiate between available sources. For instance, highly trusted sources would be public officers, newspapers, crowd-verified information, georeferenced social media posts, geolocated social media posts, or single items contributed by the crowd in a crowdsourcing campaign.

The evaluation of usefulness can be based on an automatic image classifier (see for instance Section 5, to discriminate interesting from not interesting images), or based on crowdsourcing for an assessment. A binary value can be associated in this case (0/1) or, alternatively, the confidence value of the classification procedure.

In a first pilot test with a focus group, we experimented with the following values for the above-mentioned coefficients on a set of images extracted from tweets in a case study about the 2014 Southern England floods. The values chosen for each dimension are shown in Table 1, Table 2 and Table 3. For location precision, we have chosen 0.7 for georeferenced tweets, since experimental evidence often suggests that the tweets are not referring to the precise position of the user (profile position, or the user moved away from the location where image had been taken).

The ranking algorithm classified the extracted images in the categories high (>0.79), medium (0.42–0.79) or low (<0.42) and the results were compared with the results from the focus group, obtaining a Pearson coefficient of 0.53 and *p*-value of 0.000006. Finally, a method to automatically correlate the different metrics to better model the interdependencies which exist among them is work in progress.

### 4.7. Image Classification and 3D Reconstruction from Videos

Social media networks allow referring to other users and forwarding posts and media of people in a user’s network. Therefore, the information shared is often not unique but available in multiple versions. In the first pre-processing step duplicates are detected and erased from the data set. The remaining photos are used for further interpretation. This is achieved through machine learning and related image recognition algorithms. The approach is based on convolutional neural networks that are trained with a large number of labelled images [93]. The labelling task is divided into micro tasks and carried out by the crowd. It is necessary to obtain a comprehensive database with images in disaster context but also current images of a disaster event.

**Figure 4:**
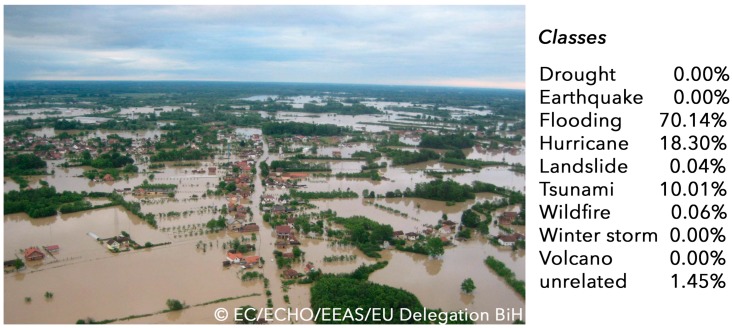
Image Recognition Interpretation Result.

In the analysis edge and shape detection as well as colour histograms are used to segment images. Through an attribution, the machine is trained and learns to interpret images with similar characteristics. For the image analysis component, Google’s TensorFlow library [94] for machine learning is used. It allows transfer-learning meaning that it is not required to develop a model from scratch but to build on a previously trained model and to develop only a new final layer. Photos of different hazard types, buildings and infrastructure will be collected through web searches and publicly available image catalogues and labelled. Figure 4 shows the outcome of a classified photo. As a result, a probability measure is provided. An additional filter can be applied to select only images exceeding a certain probability threshold.

As part of this model, the goal is to create a 3D reconstruction of a scene by applying the SfM algorithm. Videos that are gathered from social media sites are analysed for their usefulness. Selected videos will be extracted from single sequences leading to a significant number of still photos. When the camera was moving during the recording of the video, the multiple recording positions and perspectives create the required image overlap enabling the SfM processing. Different commercial and no-cost image processing tools are just being investigated for their use and the quality of their output will be compared. The resulting 3D models will be visualized through the web, as explored in [95].

### 4.8. Geospatial and Hot Spot Analysis

After a disaster, a major interest lies in determining the impacted area of the disaster and assessing the damage and the affected population. Through a spatial hot spot analysis, social media data can be clustered and visualised on a map. The classification of the social media data as well as representative coordinates for the location of the social media posts are the major requirements for this analysis. In the designed architecture, the geocrawler component (see Section 4.3) collects georeferenced social media data and the topic modelling component (see Section 4.4) extracts semantic topics from the social media data, which are then analysed geospatially to identify disaster-related hot spots.

The hot spot analysis can be used for different objectives which are related to the classification of the social media data. For example, the social media data can be classified in disaster-related and not disaster-related. This results in an overview of the exact areas affected by a disaster. Another possibility is the classification of social media data as damage-related or not damage-related. Then, the map constitutes a valuable asset for the recovery phase of the disaster management cycle. In case of disaster, the map will be continuously updated. The results will naturally depend on the availability of a critical mass of data.

For the hot spot analysis, the social media must be normalised, for instance, by the number of topic-related datasets, population density, social media data density, or a combination of the aforementioned. Ultimately, the hot spot analysis generates clusters of social media posts which are classified as relevant with a certain confidence level. The identified clusters show hot spots (high normalised density of relevant posts), which are complemented by cold spots (high normalised density of non-relevant posts). The hot spots provide a strong indication of the affected area and population, as well as the damage that has been caused.

### 4.9. Crowdsourcing: Integrating User-Generated Content from Volunteers and Experts

The increasing number of Internet users and mobile devices able to establish Internet connection allow volunteers to overcome physical distances and cooperate remotely in a very efficient manner using online platforms. Both laypersons and emergency management professionals perform useful task-based activities related to information management during a crisis. Examples of such activities are: labelling images, geotagging (adding coordinates to content), mapping (using satellite and/or reference images) and reporting damages on the field [96]. In E2mC, both experts and novices contribute valuable information for the analysis components, operators and user groups. As such, crowdsourcing is seen as a crucial part of most analysis components in the project’s architecture.

#### 4.9.1. Novice and Expert Crowdsourcing Nodes

The contribution in crowdsourcing projects from novice and expert volunteers is a common practice within emergency management efforts aiming to generate high quality data and there are multiple ways in which their different level of expertise can be integrated.

As an example, Geotag-x [97] creates tutorials for new volunteers based on experts content. Previous analysis of data quality on GeoTag-x [98] under the Citizen Cyberlab EU project [99] demonstrated the need to have tutorials for ensuring the quality of data coming from the volunteers.

A second example using experts’ contribution to improving the quality of the content generated by volunteers appears on OSM Tasking Manager [100] that is an open source platform for managing OSM contributions developed by HOT. Here, the volunteers can have different roles, giving the role of the validator to expert mappers. Tasking Manager allows to select the areas where mapping efforts are required, divide the area into subareas to prevent people mapping on the same location and analyse the evolution of the mapping in real time. At the end of the chain of volunteer activities it allows experts to validate the volunteers’ contributions before considering a task done.

#### 4.9.2. Enriching Social Media Content

The quality of social media posts varies and such content needs to be validated and classified. Additionally, often posts need further analysis to pinpoint the localization more accurately or better understand if the media or other content is useful at all. In many cases, human intelligence is needed to complement machine learning and other data processing systems in terms of retrieving the information that really matters from a set of data. One of the key objectives of the crowdsourcing component is to play a role in enriching relevant data captured on social media and other sources, ultimately providing “crisis intelligence” [101] to the various Copernicus EMS user groups.

The combination of automatic data mining techniques and crowdsourcing techniques provides two main advantages. First, it reduces the amount of data gathered or stored by the automated analysis of social media. This is achieved by filtering non-relevant data at the early stages. Second, it increases the data quality by contributing to the geolocalisation process, by solving disambiguation issues and by enriching data with useful specific metadata that is tailored to the emergency management landscape. Examples of such metadata include metadata related to origin, trust, authenticity, usefulness, textual classifications, image classification (damage, presence of debris, presence of a potential known landmark which might allow geolocalisation, etc.).

An additional relevant characteristic of the E2mC research and development component around crowdsourcing is the concept of utilising the Crowd Reporting paradigm (“Crowd as a reporter”) [96] to increase Copernicus EMS service quality and performance. Taking advantage of the presence of internet-connected volunteers (both novices and experts) within the AOI, this component allows volunteers to contribute with media information and structured information reporting the situation on the field. The perspective and motivation of these people would be that, while confronted with the disaster and its effects from a personal or professional point of view, they can personally and significantly participate in each of the four phases of emergency management by providing the Copernicus EMS service with useful data directly reported from the AOI. The advantage of such data, to be gathered on social media and on a dedicated crowdsourcing platform, would be that it can be structured immediately according to the needs of the users. Examples of such data include precise geolocation, a correct timestamp, images from different angles, etc. In comparison with analysing social media data, the ‘reporter’ scenario allows useful metadata (such as the geolocation of an image or the state of a roadblock) to be added at the time of creation, so it does not need to be derived or added by further analysis in a later stage. Compared to raw, unstructured, mostly non-geolocalised datasets that one can compose of social media posts, datasets created and contributed by volunteers are smaller yet more efficient.

Various components in E2mC’s architecture use the different kind of crowdsourcing roles (Crowd as a Sensor, Crowd as a Social Computer, Crowd as a Reporter, Crowd as a Microtasker) [96] to perform part of the tasks involved (see Section 4.4, Section 4.5, Section 4.6, Section 4.7 and Section 4.10).

### 4.10. Accuracy Assessment of Extracted Semantic Topics

The topic model components described in Section 4.4 and Section 4.5 clusters data according to its textual content. As stated in this section the primary purpose of the topic modelling is the identification of disaster- or damage-related posts. The identified topics can be interpreted with a keyword algorithm or by the crowd or the EMS operator. After the interpretation, the underlying classification accuracy must be assessed to evaluate the performance of the algorithm. Like the step before, the crowd can label social media data to assess if data is disaster- or damage-related. To overcome the subjectivity of the classification, the same data is labelled by various people. Statistics, e.g., Fleiss Kappa statistics [102] can measure the correlation between the classification of different people.

A way to decrease the required time to label social media posts is to create a labelled data set in the mitigation and preparedness phase of a disaster. This data set is added to the newly collected data in the response phase as an input to the topic models. The purpose of this is to instantly determine the accuracy of the topic model through a statistical evaluation as described in the following paragraph. This is possible as the topic model only takes the semantic of the social media posts into account.

A two-dimensional confusion matrix states the accuracy of the classification by showing the number of actual and predicted classifications [103]. The confusion matrix consists of four values: true positives (manually and automatically classified as positive), false negatives (only manually classified as positive), false positives (only automatically classified as positive) and true negative (both classified as negative). On this basis, more advanced statistical measures can be calculated precision, recall and F-measure [104]. The pre-labelled data can be created by the crowd in that several people annotate the same data sets to achieve high certainty that the data is correctly labelled.

In the next step, the topics are interpreted, i.e., the combination of words in each topic is assigned semantic meaning. The interpretation is either done manually by the operator or through a crowd tasking approach or automatically in a keyword-based algorithm. It shall be noted that one semantic topic can span across several topics extracted by LDA due to the varying combination of words over the topics.

### 4.11. Result Visualisation

The visualisation of the results of the social media and crowdsourcing analysis is a key element to ensure a smooth integration of these non-conventional data sources into the Copernicus EMS workflow. In general, the developed visualisation user interface provides tools to manage the spatial component of the social media/crowdsourcing data sets by allowing for overlaying them on a map.

There are different degrees of spatial accuracy and level of aggregation associated with the social media/crowdsourcing information layer. This problem can be tackled by using aggregated information (e.g., heat maps) to prioritize image analysis areas or satellite tasking, while media content with poor geolocation attributes coming from social media sources can also still be of use if analysed by an expert operator.

**Figure 5:**
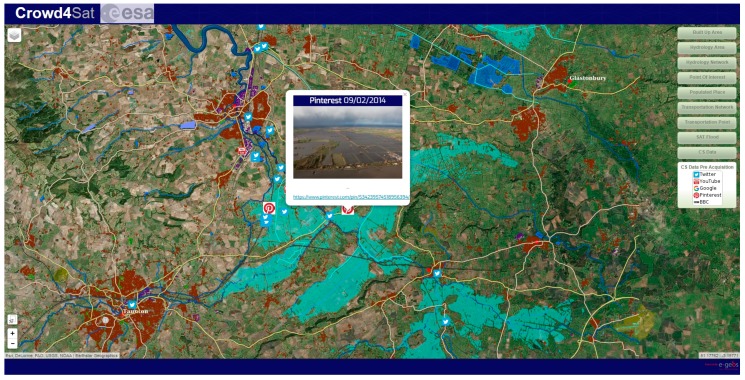
Example of a WebGIS interface showing geolocated social media data overlaid on the flood extent of the UK floods in 2014 (The activity was carried out under a programme of, and funded by, the European Space Agency) [105].

A typical example of the visualisation of the social media/crowdsourcing analysis on a map is shown in Figure 5, where a picture related to the large UK floods occurred in 2014 is extracted from Pinterest and precisely geolocated and overlaid on the flood extent derived from the pure satellite data processing.

**Figure 6:**
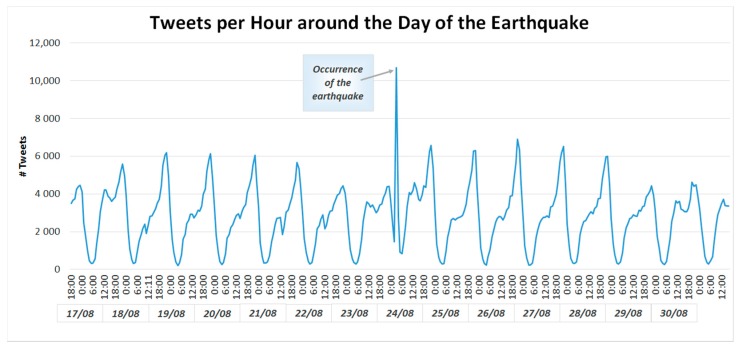
Example histogram is showing clear peaks on the frequency of tweets associated with the 2014 South Napa earthquake [74].

Other examples include the generation of dynamic heat maps which show the temporal and spatial evolution of the density of intensity of discussions on certain topics of interest (see Section 5.1). Additionally, there are also non-spatial methods of result visualisation which can still bring significant value to the Copernicus EMS workflow. For example, a simple histogram of the evolution over time of the intensity of communication, i.e., the number of relevant social media posts, associated with a group of topics of interest can be used as an effective tool for event confirmation as shown in Figure 6 for the 2014 South Napa earthquake. Similar patterns where found for the 2016 Central Italy earthquake [64], clearly showing the peaks in tweet numbers in correspondence of tremors.

## 5. Preliminary Results

The architecture presented in Section 4 has been validated in the course of a number of research efforts, delivering experimental results. The disaster analysis and management workflow has been applied to different disasters like the Napa Earthquake in 2014, the Haiti Hurricane Matthew in 2016 and the Central Italy Earthquake in 2016.

### 5.1. Combining Machine-Learning Topic Models and Spatiotemporal Analysis

On 24 August 2014, an earthquake occurred in the North San Francisco Bay with a magnitude of 6. In [74], a total of 1,012,650 georeferenced tweets were collected in the time period from 16 August 2014 to 21 August 2014. The validity of combining machine-learning methods for topic modelling (LDA) together with geospatial analysis is demonstrated in [74] where earthquake footprints and damage information from social media data were extracted. In fact, tweets could be classified in disaster-related and non-disaster-related, as well as in damage-related and non-damage-related. The results of the topic extraction were assessed with manually labelled data. In the next step, the semantic information was analysed using local spatial autocorrelation methods (Getis-Ord G_i_* [106]) for identifying hot spots. Thus, the impacted area of the earthquake could be delineated and the areas of clustered significant losses could be identified. For the latter task, LDA was applied in a cascading manner, i.e., the earthquake-related topic extracted in the first LDA iteration was again analysed using LDA to identify damage-related sub-topics. The results show that earthquake footprints were reliably and accurately identified for the given use case by validating them with the official earthquake footprint by United States Geological Survey (USGS) and the results of the HAZUS loss model.

**Figure 7:**
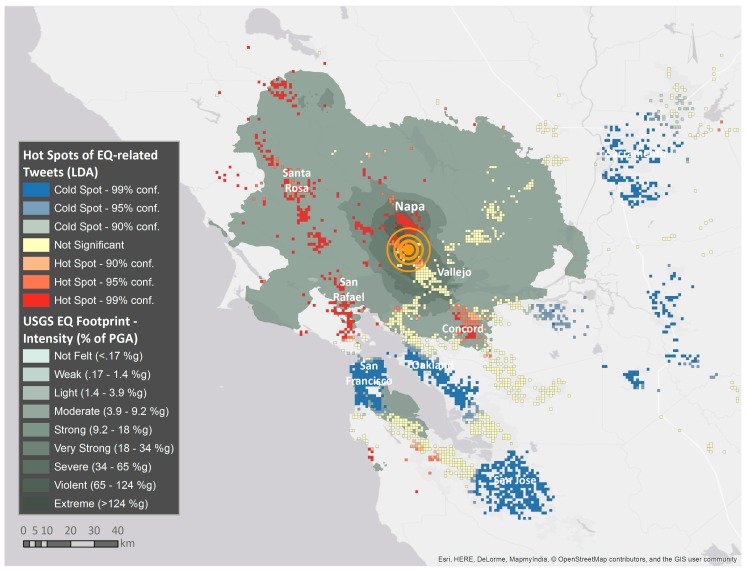
Earthquake Footprint as Identified by Analysing Social Media Posts [74].

Figure 7 illustrates the results of the earthquake footprint detection, overlaid with the USGS footprint, showing that the area and population affected by the earthquake could be accurately identified, which is also statistically validated in the paper.

### 5.2. Damage Assessment Based on User-Generated Data

Hurricane Matthew struck Haiti on 4 October 2016 as a Category 4 hurricane, causing massive destruction. About 2.1 million people were affected, including 806,000 people in need of urgent food assistance OCHA Situation Report No. 10 [107] and Situation Report No. 17 [108].

**Figure 8:**
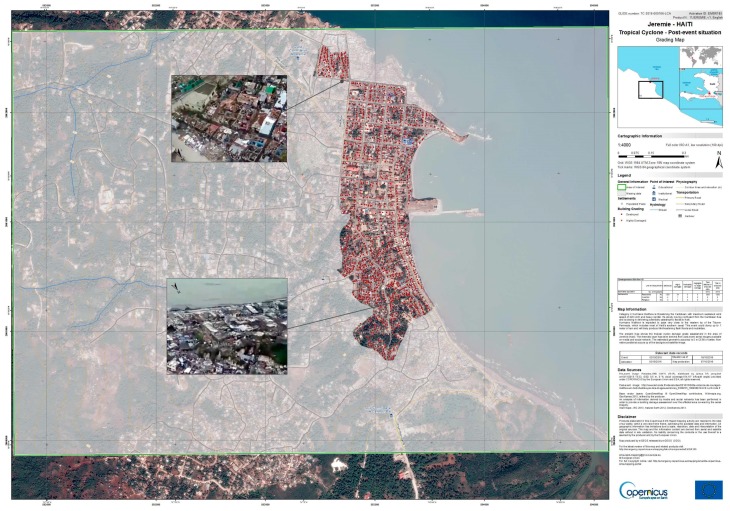
Grading map produced over Jérémie (Haiti) based on the visual interpretation of a video recorded from a helicopter distribute by Le Monde [110], Copernicus Emergency Management Service (© 2016 European Union), EMSR185.

The Copernicus Emergency Management Service (EMS) Rapid Mapping was activated on 3 October 2016 by DG ECHO ERCC [109] in order to provide timely information on damages detected through the analysis of very high-resolution satellite images over more than 40 AOI. This massive map production process started immediately after the disaster exploiting new satellite images acquired by several satellite operators and started generating first post-disaster damages assessment maps in few hours. However, persisting bad weather conditions did not allow the collection of suitable satellite images over some AOI and therefore the Copernicus EMS Rapid Mapping Team started browsing the web looking for an alternative, “non-conventional” sources of information. Such research brought relevant results of the Jérémie area in Haiti, as Le Monde online news website featured a video [110] that was collected on 6 October 2016 during a helicopter survey which was covering most of the Jérémie’s more densely populated urban area. The Copernicus EMS Rapid Mapping Team could exploit this video to assign damage classes to the single buildings which could be visually recognized both in the pre-event very high-resolution satellite imagery and in the post-event video acquired from the helicopter. Such analysis led to the production of a standard Copernicus EMS Rapid Mapping Grading Map, though with a validity of the analysis limited to the area covered by the helicopter video (see Figure 8).

### 5.3. Geolocating Social Media Posts

In a two-day time frame after the earthquake in Central Italy on 24 August 2016, a total of 152,062 tweets were collected, where a total of 26,914 tweets contained images [64]. In this case, 0.35% of the collected tweets had GPS-coordinates which shows the need for a sophisticated geotagging technique. The textual content of the tweets was compared with a keyword list that could indicate a precise geographical position. After this process, the number of tweets with explicit geolocation was increased from 533 (0.35%) to 3357 (2.20%). These tweets provide a link to 973 images that were manually associated with the tweets filtered location- and damage-related keywords. In addition to eliminating duplicates, the images irrelevant content (e.g., screenshots of tweets) were filtered. After this manual procedure 541 selected images were left, 20 of which were among the 39 images filtered by means of damage-related keywords.

Overall, the semi-automated work described has allowed to automatically retrieve 973 geolocated images with geographical coordinates, out of which 541 were potentially useful for rapid mapping purposes. Attempts to further filter these images with damage-related keywords have not proved effective as they eliminate the majority of potentially useful images and do not guarantee that remaining few images are useful.

## 6. Discussion and Limitations

The disaster management architecture using social media and crowdsourcing techniques, as described above, results in a number of limitations. The data themselves contain various uncertainties like inaccuracy of GPS-coordinates, underrepresented communities, etc. but also the analysis methods, reliability of volunteers or the companies behind the social media networks add to the uncertainty.

### 6.1. Limitations of Social Media Data

The limitations of social media data can vary between social media networks, e.g., the semantic meaning is easier to identify in posts of social media networks such as Facebook in comparison to Twitter posts, which is mostly due to the limited number of characters for tweets. However, there are multiple limitations in terms of spatiotemporal and semantic information that are present in social media posts from Twitter, Flickr, Facebook and Foursquare. Most of these limitations are covered in previously published literature [111,112] and, therefore, are only shortly described in this paper.

The georeferenced information of social media posts can be extracted from various kinds of information, e.g., GPS coordinates, a manually entered place when posting a post, or the user’s profile location. The accuracy of the GPS coordinates can be low and the user defines the location by itself, which biases the results. Furthermore, not all of the people are participating in social media networks and so the results of the analysis are targeting only a specific group. Furthermore, bots are more and more present in social media, spamming the network with automatically produced posts that are not relevant for any analysis method covered in this paper.

The semantic content of the social media data implicitly reflects the user’s environment and mental states and can thus sometimes be difficult to interpret. The interpretation becomes even more challenging if the user’s post refers to the past or the future. A further limitation is the use of informal language in social media networks, including slang words, abbreviations, emoticons, irregular punctuation, or youth speak. This limitation can be overcome by extensive data pre-processing but which may lead to information loss.

### 6.2. Spatial Hot Spot Analysis

For the hot spot analysis, a grid needs to be created, which may compromise the accuracy of the results because data sets are spatially aggregated into raster cells, causing a modifiable area unit problem (MAUP) [43]. In fact, the chosen cell size, as well as the placement of the grid, can influence the results of the hot spot analysis.

Another problem is the heterogeneous temporal progression of different disaster types: While floods are spreading across space and cause a slowly increasing activity in the social media networks, earthquakes hit an entire area without early indications, which usually leads to an abrupt peak in social media activity. Thus, the methods for detecting and analysing may differ between various disaster types.

Another drawback is induced by the dependency of the geospatial analysis on the topic extraction mechanism. If an AOI is geographically large, smaller topics may be hidden in the mass of identified topics. This effect is, even more severe in densely populated urban areas. Therefore, small-scale events have a lower probability of being detected.

### 6.3. Crowd Management

Crowdsourcing communities can potentially provide powerful support capabilities for disaster management [113]. However, such groups are mostly unpredictable and highly volatile in nature in the sense that they are based on the voluntary engagement of their members, many of which are to be recruited during actual activations, in rapidly changing circumstances. Therefore, it is difficult to anticipate the number of people that will join and when and for how long each of them is willing to help.

There are many factors involved in the decision of person, be it a layperson or expert, to offer technical skills or other individual resources to a crowdsourcing initiative. Based on existing studies on motivators for citizens [114] and emergency management experts, the E2mC crowdsourcing component tries to maximize volunteers engagement by considering the following activities [115,116]:

First, provide highly intuitive platforms, systems and processes and systematically provide value and recognition in return for the contributions done.

Provide preparation activities during the periods when the crowd is not in its activated mode (skill training, capacity building activities, or physical gatherings are just a few examples). This keeps the initiative “top of mind” among its members and generates increased engagement when an actual activation occurs. Furthermore, such activities allow crowd members to maintain and improve their skill levels for the various tasks, improving the quality of the end product.

Build and maintain a core team of volunteers who actively recruit new members before, during and after activations. These core persons are also prepared for a broad and wide campaign at the point when a crowd activation occurs, when the momentum generated by news reports will attract a lot of new volunteers.

Build strong relationships with external parties and communities, both the ones involved in crowdsourcing activities and those that will use the results of the activities, i.e., related to different aspects of disaster management.

Support and promote the community not as a faceless network but as a broad group of engaged citizens and experts from various backgrounds who can interact and learn from each other.

### 6.4. Crowdsourced Data Quality

In any crowdsourcing project, the data contributed by volunteers come with varying quality due to the heterogeneity of contributors, using various technologies and tools and with different level of expertise in performing the required actions. When it comes to Volunteered Geographic Information (VGI), several methods have been developed to assess and improve the quality of data and a complete overview is provided by [117].

A way to assess and describe quality is based on the concepts of quality measures and quality indicators [118]. Quality measures are mainly obtained by comparing volunteers’ data with official one and include aspects of completeness, consistency, positional accuracy, temporal accuracy and thematic accuracy.

However, traditional comparison is not viable in every application scenario, especially in the case of natural disasters (lack of reference data). When data are not available for comparisons, researchers have explored quality indicators that rely on various participation biases, contributor expertise or the lack of it, background, etc. Data quality has also been shown to depend strongly on the form of information like map, image, text and the context of usage [117]. Three approaches are propose in [119] to ensure the quality of VGI data: crowdsourcing (group validation), social (reputation and gatekeeping) and geographic (comparison). Issues of data credibility for contributions coming from the crowd have been addressed amply in the context of Citizen Science projects, where date are collected and and/or analysed by non-professional [98,120,121,122,123,124,125].

Many of these issues can be overcome by using statistical methods of data analysis on the multiple independent interactions with the data (‘wisdom of crowds’), allowing to understand the accuracy of the data [120]. Other accepted solutions to improve data quality include: Training, even via a simple tutorial, can reduce the variability between volunteers [126]; Cross-checking for consistency with their own observations. Then, volunteers can learn to eliminate bias through training and experience [127]; Quiz-style questionnaire at the end of surveys; Simplifying the tasks asked of the public and/or adapting the research questions. A frequent assumption is that volunteers collecting data are unable to follow complex scientific protocols [128]. However, comparative studies have demonstrated that volunteer capabilities can be equal to professionals (e.g., [129,130]).

One more solution is related to the concept of gamification. Due to the clear incentives of this data collection approach (going high up in rankings, collecting badges, etc.) this popular method can be used to control the process of collecting more accurate data by incorporating data quality concepts [131]. One way to do that would be to give a ranking to the contributor based on the quality of their collected data. Revealing such rankings of their peers would further encourage the contributors to pay more attention to the quality of their data (peer pressure).

### 6.5. Integration with Remote Sensing Based Information

The integration of social media and crowdsourcing analysis with remote sensing (satellite and non-satellite, for example, aerial or images taken by drones) has enormous potential as demonstrated throughout this paper. However, there are also important limitations which have to be properly analysed and taken into account for future operational integrated services.

**Availability**: availability of social media and crowdsourcing data highly depends on external factors such as the level of penetration of the use of social media in the local population, which varies significantly at country and city level, the data policies of private companies owning and operating social media networks like Twitter and Flickr and the availability of crowds committed to contributing to the requested tasks (e.g., mapping, tagging, filtering, translating). Furthermore, the destroyed infrastructure in the affected area may reduce accessibility to social media networks, which again results in less activity in the networks when it matters most.

**Timeliness**: timeliness of social media analysis results and crowdsourcing contribution is a key element as it has to be consistent with the requirements set by the Copernicus EMS workflow. In particular, such social media data have to be available within very few hours after a relevant event has occurred, as they need to act both as a timely trigger for fresh satellite images collection and as gap filler before such images are delivered. Additionally, crowdsourcing contributors have to be ready to be mobilised at short notice in order to provide an effective support in massive map production tasks and, in case of crowds of locally affected people, they should be ready to provide timely in field feedback/report to be used as in situ truth during the satellite image interpretation process.

**Spatial accuracy**: spatial accuracy of the derived content of social media posts and crowdsourcing analysis is a relevant parameter and main driver of the integration process between social media and remote sensing data. Even though social media data are still valuable even with coarse geolocation information, the higher geolocation accuracy opens up new opportunities for the automatization of the joint analysis of social media data in combination with remote sensing images. Effective ways to improve geolocation accuracy of social media contents have to be explored (for example, contacting the originator of the social media content in order to get precise geolocation information).

## 7. Conclusions and Outlook

This paper sketches the developed system architecture of the EMS Witness component and describes the new functionalities of the EMS service in the form of scenarios and presents several preliminary case studies, providing evidence that the scientific and operational goals have been achieved. The architecture consists of several newly developed components: Geocrawler, keyword-based crawler, text, image, video and geospatial analysis, content geolocation, accuracy assessment of text analysis, crowdsourcing integration and result visualisation.

The presented architecture goes beyond the state of the art with respect to several aspects. First of all, it combines a variety of cutting-edge research methods into a single architecture, as sketched out in the previous paragraph.

Furthermore, the presented system provides information in the first hours of an event, which is achieved by producing maps and indicating the impact and the damage of the disaster based on user-generated data. The added information layer consists of hot spots analysis of various stages of the event as well as images of the disaster in the AOI.

In addition, user-generated data from social media and targeted crowdsourcing activities are combined with remotely sensed data, in particular satellite imagery. In fact, in-situ information from images and crowdsourced textual descriptions of the disaster can be assimilated to ground truth and can then be exploited to enhance satellite image analysis and interpretation. After the critical time period of the disaster the new information system is continuously updated by the analysis of social media and crowdsourced data and can orient the satellite tasking through the complete task.

Furthermore, the system enhances the usability for the operator and for professional users as it is equipped with multiple user interfaces and data management and storage components. As a result, the Witness component adds new information in almost all phases of the disaster management cycle to the EMS by meeting the following criteria. In the preparedness phase, the component indicates a new relevant crisis event and can confirm information created by EWS. This enables operators to activate satellite tasking before (or immediately after in case of earthquakes) an event takes place.

Other benefits of the new system are the vast amount of information contributed with human intelligence via the social media networks and a web application for preselected people. The EMS portfolio is enriched by a new product that increases the production capability of the system, complementing the bulk analysis and interpretation of satellite images through crowdsourcing with quality control procedures implemented by Copernicus EMS providers. Ultimately the citizens are involved in the process of helping authorities during a disaster that increases the awareness of Copernicus and enlarges the service usage. In the mid-term future, the architecture will be integrated into the EMS workflow. Then, the component functionalities can be tested and applied in real-world scenarios to verify the propagated goals with experimental results. The main challenge will be to reduce the execution time of workflow to its minimum so that the output is generated in near real time.

## Figures and Tables

**Table 1:** Location precision coefficients.

Location Precision	Value
Street level or exact position of POI	1
Georeferenced social media post	0.7
Locality level	0.67

**Table 2:** Trust of source coefficients.

Trust of Source	Value
Public officer	1
Newspaper or journalist	0.8
Any user	0.6

**Table 3:** Usefulness coefficients.

Usefulness	Value
Possibly useful (even if not certain)	1
Not useful	0
